# Imiquimod does not elicit inflammatory responses in the skin of the naked mole rat (*Heterocephalus glaber*)

**DOI:** 10.1186/s13104-020-05260-6

**Published:** 2020-09-05

**Authors:** Mosiany Letura Kisipan, Rodi Omondi Ojoo, Titus I. Kanui, Klas S. P. Abelson

**Affiliations:** 1 Department of Veterinary Anatomy & Physiology, Egerton University, Egerton, P.O. Box 536, Nakuru, 20115 Kenya; 2 Department of Veterinary Anatomy & Physiology, University of Nairobi, P.O. Box 30197, Nairobi, 00100 Kenya; 3 Department of Agricultural Sciences, South Eastern Kenya University, P.O. Box 170, Kitui, 90200 Kenya; 4 Department of Experimental Medicine, University of Copenhagen, Blegdamsvej 3B, 2200 Copenhagen, Denmark

**Keywords:** Naked mole rat, Imiquimod, Skin, Inflammation, Psoriasis, Negative model

## Abstract

**Objective:**

Naked mole rat (*Heterocephalus glaber*) has recently attracted interest in biomedical research due to its exceptional longevity, cancer resistance and tolerance to potentially harmful conditions or stimuli. Given its unique attributes, this study was designed to characterize inflammatory skin reactions of this animal to topical application of imiquimod, a toll-like receptor 7 and 8 agonist that triggers psoriasis-like skin reaction.

**Results:**

Imiquimod did not cause the expected psoriasis-like skin changes. There was no epidermal thickening and a straight epidermo-dermal boundary was maintained. There was no parakeratosis and the granular layer of epidermis was well formed. In the dermis, there was no leukocyte infiltration. This points to an exceptional nature of inflammatory/immune responses of this animal, but the mechanism could not be explained by our results. Naked mole rat could be a valuable negative model for studying psoriasis and other inflammatory skin conditions but as a prerequisite, there is need for further investigations to establish the mechanisms behind its lack of response to imiquimod.

## Introduction

The naked mole rat, NMR (*Heterocephalus glaber*), is a Bathyergid rodent distributed in the hot and dry regions of the horn of eastern Africa [1, 2]. NMRs are unique in in that they are eusocial, poikilothermic, and exhibit extreme longevity with minimal signs of aging [1, 3–6]. The skin of this animal is loosely folded over the body and lacks pelagic hairs, hair follicles, sweat and sebaceous glands [7]. The junction between epidermis and dermis is regular with no epidermal ridges nor dermal papillae and the melanocytes are found within the dermis.

Psoriasis is a chronic immune-mediated inflammatory skin disease with strong genetic predisposition and a high prevalence in adults than in children [8–12]. It is characterized by well demarcated erythematous, indurated and pruritic skin plaques covered by silvery scales [9, 12–14]. Though predominantly a skin disease, psoriasis is associated with arthritis, cardiovascular diseases and other comorbidities [9, 14, 15]. Histopathological features of psoriasis include a thickened epidermis (acanthosis) due to increased keratinocyte proliferation with loss of granular layer, thickened stratum corneum (hyperkeratosis) and retention of nuclei in outer layers of epidermis and stratum corneum (parakeratosis). There are also collections of neutrophils in epidermis including stratum corneum referred to as Munro’s microabscesses. In the dermis, there is dilation and increase in blood vessels and infiltration of inflammatory cells [9, 16, 17].

Imiquimod (IMQ) is a toll-like receptor (TLR) 7 and 8 agonist used topically to treat external genital and peri-anal warts caused by human papilloma virus, actinic keratosis and superficial basal cell carcinomas and potential to treat many other skin disorders [18–26]. Through activation of its receptors, IMQ application, even on a short-term basis, triggers inflammatory skin reactions that closely resemble those seen in psoriasis [27–30]. The use of this regimen in mice has since been preferred as a model for studying the condition [30–34].

NMRs resist or tolerate various potentially harmful conditions or stimuli including hypoxia [35], inflammatory pain [36], histamine-induced itch [37] and cancer [38–40]. Due to these traits and its extreme longevity, the animal has attracted a lot of interest in biomedical research [41]. Given its resistance and unique skin structure, the animal could be a good model to study psoriasis and other skin disorders.

This study was designed to characterize the responses of the skin of NMR to topical application of IMQ by describing both the macroscopic and histological changes as a step in exploring whether the animal could be a model for studying psoriasis and other skin conditions.

## Main text

### Materials and methods

#### Animals, husbandry, and study design

A total of 22 NMRs from a colony captured from Kibwezi in Makueni County, Kenya, were kept in a dark temperature-controlled room at the main campus of the South Eastern Kenya University (SEKU). The animals were housed in Perspex cages with interconnected compartments and wood shavings as bedding material. After three weeks of acclimatization, the animals were randomly re-distributed to four groups of 5–6 animals housed in separate cages and then randomly assigned to different treatments as shown in Table 1. No power analysis was made for group size determination. Given the nature of the study, a group size of 5–6 animals was considered sufficient and supported by the Resource Equation Method [42]. A further three weeks acclimatization period was allowed before the start of the treatments. In brief, two groups, which served as controls, received topical Vaseline over the shoulder region (Group I) or over the rump (Group II). The other animals received topical IMQ (Aldara™ 5% cream; 3 M Health Care Limited, United Kingdom), which was also applied over the shoulder region (Group III) and over the rump (Group IV). Vaseline was applied once daily at 09:00 h for eight consecutive days while imiquimod was applied twice daily at 09:00 h and 16:00 h for eight consecutive days as summarized in Table 1. The duration of testing was based on previous studies in mice [32, 33]. All test agents were applied on the skin on an area approximately 1 and 2 cm^2^ respectively over the shoulder and rump. In all animals, the area of application was examined daily for any changes. At the end of the treatments, the animals were anesthetized using isoflurane immediately followed by euthanasia by decapitation.

**Table 1:** Summary of the study design

Groups	n	Time of application																	
		Day 1		Day 2		Day 3		Day 4		Day 5		Day 6		Day 6		Day 7		Day 8	
		9:00	16:00	9:00	16:00	9:00	16:00	9:00	16:00	9:00	16:00	9:00	16:00	9:00	16:00	9:00	16:00	9:00	16:00
I	6	VAS	–	VAS	–	VAS	–	VAS	–	VAS	–	VAS	–	VAS	–	VAS	–	VAS	–
II	6	VAS	–	VAS	–	VAS	–	VAS	–	VAS	–	VAS	–	VAS	–	VAS	–	VAS	–
III	5	IMQ	IMQ	IMQ	IMQ	IMQ	IMQ	IMQ	IMQ	IMQ	IMQ	IMQ	IMQ	IMQ	IMQ	IMQ	IMQ	IMQ	IMQ
IV	5	IMQ	–	IMQ	–	IMQ	–	IMQ	–	IMQ	–	IMQ	–	IMQ	–	IMQ	–	IMQ	–

*VAS* Vaseline, *IMQ* imiquimod

#### Samples collection, processing, and examination

Full thickness skin from sites where topical test agent was applied was dissected out, fixed with 10% buffered formalin, processed for wax embedding then sectioned, mounted on glass slides and stained with hematoxylin and eosin. Coverslips were applied using DPX mountant. The mounted sections were examined by two blinded examiners using a Leica DM 500 light microscope connected with a Leica ICC 50 digital camera (Leica Microsystems, Wetzler, Germany).

## Results

**Fig. 1:**
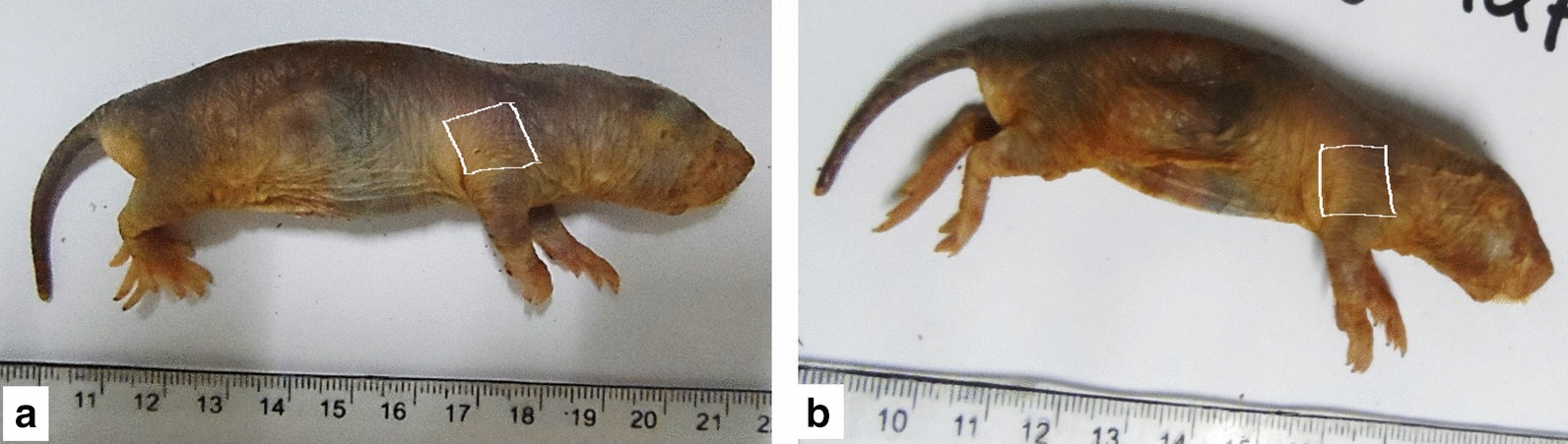
NMR that received topical Vaseline (**a**) and IMQ (**b**) over the shoulder region (under the area marked with the white line). IMQ did not induce any changes in the skin. Calibrations are in centimeters
ii) *Histopathological findings*
  The epidermis did not show any thickening in all the groups, and a straight boundary between the epidermis and dermis was maintained with no rete ridges (Fig. 2 and Additional file 2: Figure S2). No parakeratosis or Munro’s micro-abscesses were present, and the granular layer was well formed. In the dermis, there was no vascular congestion nor leucocyte infiltration.

i) *Macroscopic IMQ response*
  No signs of erythema, scaling or any other skin changes were observed on the area of the skin where Vaseline or IMQ was applied (Fig. 1 and Additional file 1: Figure S1).

**Fig. 2:**
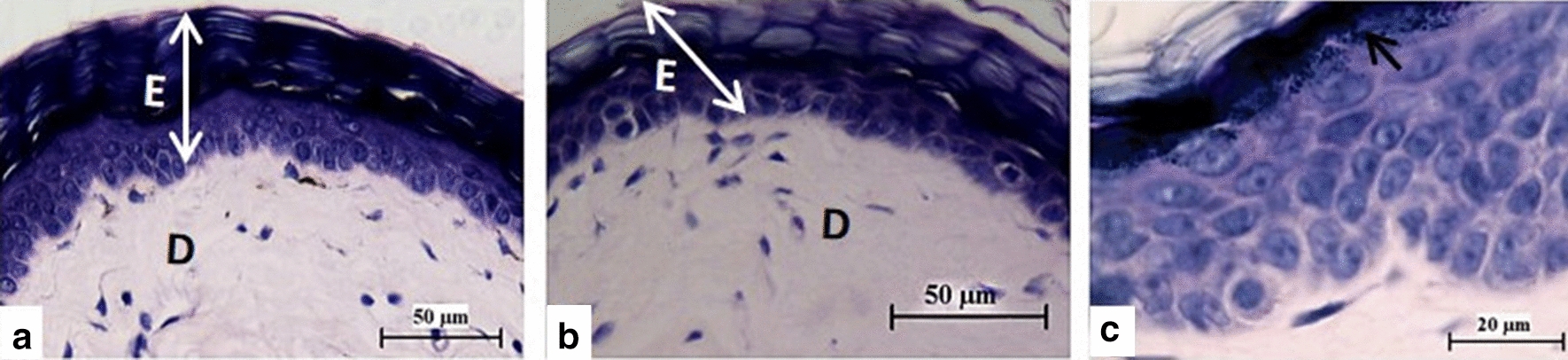
Histological sections of NMR skin from the shoulder region obtained after topical application of Vaseline (**a**) and imiquimod cream (**b**) for eight days. There are no changes in the skin structure after application of Vaseline or IMQ, with no leucocyte infiltration nor vascular congestion and the straight boundary between epidermis (**E**) and dermis (**D**) is maintained. Epidermis of NMR that received topical IMQ for 8 days (**c**) showing a well-formed stratum granulosum (**arrow**)

## Discussion

Topical application of IMQ elicits an inflammatory reaction in murine skin characterized grossly by erythema and scaling. Histologically, the features include epidermal thickening, altered epidermal differentiation with retention of nuclei in stratum corneum, absence of epidermal granular layer and leukocyte infiltration in the dermis [31–33]. These features closely resemble human psoriasis so well that this regimen has been suggested as a fast and reproducible model of studying the condition [31, 43]. The effects of IMQ are mediated by TLR 7/8 which are mainly expressed by monocytes, macrophages and dendritic cells [31]. Studies have also suggested that IMQ also act on keratinocytes either through TLR 7 [44] or a TLR-independent mechanism [45].

The results of this study suggest that the skin of NMR shows neither TLR-mediated nor TLR-independent effects of IMQ. This finding is intriguing and affirms the uniqueness and possibly the exceptional nature of inflammatory/immune response of this animal. Recent findings have shown that innate immunity of NMR possesses a number of differences, in terms of cellular composition, from what is generally known in mammals [46]. It is therefore probable that the inflammatory/immuno-surveillance components/mechanisms in the NMR epidermis that would initiate IMQ-induced skin reactions are different from that of other mammals. NMR may either be possessing, within the epidermis, atypical Langerhans cells and/or plasmacytoid dendritic cells that lack the mechanisms that mediate IMQ responses. These cells are thought to form the first line of contact with IMQ via their TLR 7/8 thus mediating the effects [47, 48]. Future studies aiming at immunohistolocalization of Langerhans cells and TLR 7 in the NMR skin may clarify this. Although a clear explanation to the non-reaction of NMR skin to IMQ could not be adduced, our results affirm the exceptional and unique nature of NMR inflammation/immunity. Furthermore, inflammation has been established as a hallmark of cancer [49–51] and it is therefore likely that the unique inflammatory/immunosurveillance mechanism exhibited by this animal could also be a contributor to its resistance to cancer.

In studies using mice, IMQ was applied on shaven area over the dorsum of the animal. Shaving before application could be significant as it has been suggested to enhance percutaneous absorption of substances [52, 53]. Being hairless, neither shaving nor sham-shaving was performed before IMQ application in the NMR. This could therefore mean that in the study using NMR, IMQ was applied to a more intact barrier and therefore the access to the cells that mediate the IMQ effects was limited if not eliminated. Furthermore, the NMR is thought to have a thicker epidermis and stratum corneum [7]. It is also possible that in this animal, Langerhans cells are localized in the dermis rather than in epidermis. This makes the cells virtually inaccessible to the ligand (IMQ) for their TLR 7/8. Such an atypical distribution of epidermal cells to the dermis in this animal has been reported in regard to the melanocytes [7].

Reactive oxygen species (ROS) are reported to play a protective role against immune mediated diseases. Increasing ROS has been shown to attenuate IMQ-induced psoriatic dermatitis while administration of anti-oxidant, N-Acetylcysteine, aggravated the condition [54]. NMR has poor antioxidant capacity, a more pro-oxidative cellular environment and higher levels of oxidative damage to macromolecules as compared to age-matched mice [55–57]. It is possible that there are high ROS in the skin of NMR which in turn confers protection against IMQ-induced psoriasis-like inflammation. This hypothesis, however, needs to be backed by further studies to analyze antioxidant capacity, oxidative damage and pro-oxidative status of NMR skin.

In conclusion, topical application of IMQ does not induce psoriaform skin inflammation in the NMR. This could be due to a combination of various unique attributes of the animal’s skin structure and/or its inflammatory/immunosurveillance apparatus. The cells that interact with IMQ and mediate its effects may have atypical distribution such that access to IMQ is limited or they could also be lacking TLR 7/8 and other mechanisms that mediate IMQ effects. High ROS reported in NMR tissues could also be exhibited by the skin and thereby compounding its resistance or tolerance to IMQ. The animal could be a good negative model for studying psoriasis but as a prerequisite, further studies are necessary to characterize the mechanisms behind the non-reaction of the animal’s skin to IMQ.

## Limitations

This study was designed to characterize inflammatory responses of NMR skin to topical IMQ. Unexpectedly, the results turned out negative. It is our strong opinion that this observation is significant and of great scientific value. Naturally, this would have been followed by further experiments to explain in detail the mechanism behind non-response. Unfortunately, resources needed to fulfil this are not immediately available. It is therefore important that this information is shared with the scientific community to stimulate further research that will build on our results through collaborations or otherwise.

## Supplementary information

**Additional file 1: Figure S1.** Photographs of NMR. There were no changes in the skin after application of either Vaseline (a) or imiquimod (b). These agents were applied in the region bound by dotted line.

**Additional file 2: Figure S2.** Histological sections of NMR skin from the rump region from animals that received topical application of Vaseline (a**)** and imiquimod cream (b). There are no changes in the skin structure after application of Vaseline or IMQ for 8 days, with no infiltration by leucocytes and maintenance of a straight boundary between epidermis (E) and dermis (D).

## Data Availability

All the data and results generated and analyzed have been presented in this published article and its supplementary information files.

## Abbreviations

NMR: Naked mole rat
IMQ: Imiquimod
TLR: Toll-like receptor
cm^2^: Square centimeters
ROS: Reactive oxygen species
