# Supplementary figures and images for: Imiquimod does not elicit inflammatory responses in the skin of the naked mole rat (Heterocephalus glaber)
Source: BMC Res Notes. 2020 Sep 5;13:416. doi: 10.1186/s13104-020-05260-6 (PMC7487461; doi:10.1186/s13104-020-05260-6)

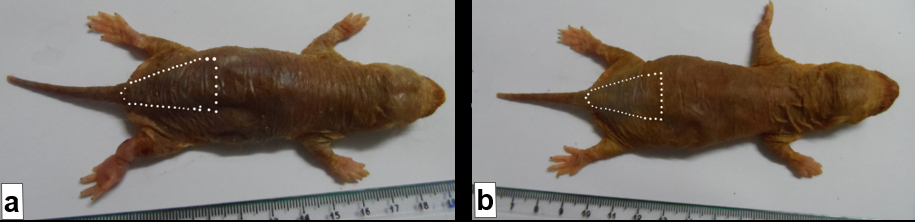

Supplement: Supplementary file 1 — Additional file 1: Figure S1. Photographs of NMR. There were no changes in the skin after application of either Vaseline (a) or imiquimod (b). These agents were applied in the region bound by dotted line. [file 13104_2020_5260_MOESM1_ESM.jpg]

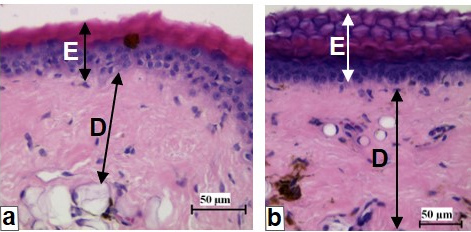

Supplement: Supplementary file 2 — Additional file 2: Figure S2. Histological sections of NMR skin from the rump region from animals that received topical application of Vaseline (a) and imiquimod cream (b). There are no changes in the skin structure after application of Vaseline or IMQ for 8 days, with no infiltration by leucocytes and maintenance of a straight boundary between epidermis (E) and dermis (D). [file 13104_2020_5260_MOESM2_ESM.jpg]
